# Home-Grown School Feeding: Implementation Lessons From a Pilot in a Poor Ethnic Minority Community in Vietnam

**DOI:** 10.1177/03795721221088962

**Published:** 2022-04-26

**Authors:** Sabina Di Prima, Dai Nguyen Dinh, Demi D. Reurings, E. Pamela Wright, Dirk Essink, Jacqueline E. W. Broerse

**Affiliations:** 1 Vrije Universiteit Amsterdam, Amsterdam, the Netherlands; 2 Guelph International Health Consulting, Amsterdam, the Netherlands; * Sabina Di Prima and Dai Nguyen Dinh contributed equally to this work and qualify as first authors for this publication.

**Keywords:** nutrition-sensitive agriculture, remote areas, preschool, school meal diversity, social entrepreneurship

## Abstract

**Background:**

Undernutrition threatens the health and future of preschool children in disadvantaged remote communities. Home-grown school feeding (HGSF) in nursery schools could positively impact children’s nutrition while creating multiple benefits for the whole community. However, evidence is lacking on implementation of HGSF within multi-sectoral programs in remote areas.

**Objective:**

This study assessed an HGSF pilot intervention, part of a nutrition-sensitive agriculture (NSA) program, in a mountain ethnic minority community in Vietnam. It aimed to identify the changes brought about by the intervention, in particular diversity of children’s food, food sources, barriers and facilitators to change, and future challenges and strategies.

**Methods:**

Mixed-methods assessment covered school meal diversity, cost, and food sources but the key focus was on observed changes resulting from the HGSF intervention and perceived barriers and facilitators to its implementation. Data were collected mainly through semi-structured interviews (n = 30) and seven focus group discussions (n = 76).

**Results:**

School meals contributed to increasing diversity of food consumed by children. Above 30% of foods used were home-grown. Respondents reported increased school attendance; children’s food preferences and hygiene practices improved as did parents’ caring and feeding practices. Local food systems became less cash-crop-oriented and more self-reliant, contributing to household food security and income generation. Social capital increased. Positive changes were attributed to HGSF and synergy among NSA program components. Poverty and limited resilience to external shocks threatened sustainability.

**Conclusions:**

Implementing HGSF within an NSA program in a mountainous ethnic minority area with a high prevalence of undernutrition benefitted children and their communities.

## Background

Undernutrition continues to be a public health problem in many countries, especially for young children and women of reproductive age.^1,2^ Recent estimates suggest that, globally, 149 million children under five years of age (CU5) are stunted and 49 million are wasted^3^; about 16 million are affected by both stunting and wasting, contributing to increased risk of mortality.^2^ In low- and middle-income countries (LMICs), children from disadvantaged ethnic minority groups are often at higher risk; on average their rates of stunting are 2.8 and of wasting six times higher than those of more advantaged peers.^1^

The causes and consequences of childhood undernutrition are multidimensional and interrelated.^4-6^ Undernutrition in the early stages of life undermines the cognitive and physical development of children with detrimental long-term consequences on their educational, productive, and reproductive performance as adults.^7-10^ Given its multifaceted nature, the undernutrition problem requires integrated solutions that involve the collaboration among multiple sectors and synergy between nutrition-specific and nutrition-sensitive approaches to address simultaneously the direct (health and diet) and the underlying (food security, health services, care practices, hygienic living environment) determinants of children’s nutritional status.^6,11-13^ Nutrition-sensitive approaches, particularly food-based approaches such as nutrition-sensitive agriculture (NSA), could play an important role by addressing the underlying determinants through context-dependent, non-linear paths^6^ with agriculture as the key entry point, collaborating with other sectors such as health and education.

In the past decades, child undernutrition has become a policy priority on national and international agendas, exemplified by the “Decade of Action on Nutrition” and the 2030 Agenda for Sustainable Development.^14,15^ The 2008 Lancet series highlighted the importance of focusing on the 1000-day period between conception and the first 24 months of life, considered a critical window of opportunity to influence the growth and development of a child.^4,10,16^ However, taking into account the nutrition and development needs of individuals across their life cycle, less critical but still important windows may appear in the following 7000 days, from early childhood^17^ to late adolescence.^18-20^ In the first 1000 days, the health system is conventionally the main agent for interventions to improve the nutritional status of infants and young children. Later, school-based nutrition programs often focus on primary school children, but preschool programs, in kindergartens or nursery schools, could also be important for children between three and five years,^15,18^ who might otherwise be neglected.

Schools can offer a suitable environment for the promotion and development of healthy eating habits.^21^ They can stimulate changes in children’s diet and eating behaviors by providing food and time with peers.^21^ Receiving meals at school could lead to increased school enrollment, attendance, and retention.^22,23^ Investing in the formative years at school and preschool could offer strong returns in adulthood.^24^ Furthermore, schools represent an appropriate (complementary) platform for multi-sectoral nutrition-sensitive interventions,^25,26^ particularly those targeting children normally beyond the reach of regular school programs, such as ethnic minority children aged 3 to 5 years in remote communities.

Globally, school feeding programs provide meals to about 368 million children from pre-primary to secondary school.^26^ Since 2003, an increasing number of governments in LMICs have started sourcing part of the food for school feeding from local farmers, with the additional objectives to improve smallholders’ livelihoods, strengthen the resilience of local food systems, promote nutrition-sensitive value chains, and reduce food waste.^15^ This approach, labelled home-grown school feeding (HGSF), facilitates the transition from external donor-driven to sustainable country/community-owned school feeding programs.^27^ Home-grown school feeding potentially offers context-appropriate solutions by providing school children with nutritious meals that fit local taste preferences^15^ and food production.^28^ Linking school feeding to local food production systems can help create a stable and structured market for local farmers, traders, and caterers in the school feeding supply chain.^15^ Decentralizing food procurement to community caterers, as social microenterprises, may increase community involvement and support, which can be important for sustainability.^15^ Because social entrepreneurship focuses on the potential of individuals and communities rather than on external help,^29^ it fits the needs in remote and hard-to-serve areas.

To our knowledge, lessons from the implementation of HGSF in nursery schools are still limited, as existing studies focus mostly on primary schools.^30-33^ Home-grown school feeding can be implemented in the context of a comprehensive package of multisector interventions addressing multiple needs.^15^ However, as Reinhardt and Fanzo^6^ remarked about the inclusion of nutrition-sensitive interventions in larger multi-sectoral programs, little is known on how HGSF components contribute to and interact with other interventions in complex NSA programs.^32^ Questions regarding benefits and costs of HGSF interventions^32^ and their implementation and sustainability in mountainous areas are yet to be answered.

These knowledge gaps about the role of HGSF in addressing undernutrition among CU5 are particularly relevant in the context of countries such as Vietnam, with rapid economic development, but where the significant improvements in reducing undernutrition are not distributed equally in the population.^34^ In Vietnam, disadvantaged ethnic minority communities in remote mountainous areas, constituting 14% of the Vietnamese population (about 13.5 million people),^34^ are most affected.^35^ Their undernutrition is exacerbated by a complex mix of agro-ecological, socio-economic, and cultural factors.^34,36,37^ Children under five years of age are particularly vulnerable, having high rates of stunting (31.4%) and underweight (21%) compared to ethnic majority Kinh children (15% stunted, 8.5% underweight),^34,38^ clearly showing the social and economic relevance of undernutrition.

We assessed the experience of an HGSF pilot intervention in nursery schools in a mountain ethnic minority community in Vietnam. In this study, we aimed to identify: (1) the changes brought about by the intervention, in particular diversity of children’s food and food sources; (2) barriers and facilitators to the change process; and (3) future challenges and strategies. The results will contribute to the evidence base on HGSF not only in Vietnam but in other low-income remote communities.

## Methods

### Intervention and Study Setting

Through two consecutive National Nutrition Strategies (2001-2010 and 2011-2020), the Vietnamese Government has prioritized disadvantaged areas and groups at high risk, promoting multi-sectoral and community-based initiatives, including nutrition programs in nursery schools, and taking a preventive approach, “Nutrition throughout the Lifecycle”.^39,40^ Aligned with this approach and the national action plan “Zero Hunger by 2025”, a four-year NSA program was implemented in the mountainous commune of Phu Mo, Dong Xuan district, the most remote and poor commune in Phu Yen province. Table 1 summarizes the situation in Phu Mo commune prior to beginning the program. The data come from the baseline study conducted by the NSA program in March 2018, on a sample of 224 households (unless otherwise indicated).

**Table 1.** Phu Mo Commune Baseline Situation.

Topics	Baseline results
Food insecurity	Based on the Food Insecurity Experience Scale, surveyed households included 13% food secure, 42% mildly food insecure, 39% moderately, and 6% severely
Children’s nutritional status	Among all CU5 (n = 243), 43% were underweight and 61% were stunted
Income earning	Overall, 87% of the households relied on labor as hired farmers, planting and harvesting cassava and acacia Cash-crop production played a central role; 46% of households earned income from cassava, with price fluctuating over time but steadily declining since 2012
Production system	Many households grew rice in wet paddy (33%) or upland (31%) fields; 96% of rice production was for own consumption About half of households raised chicken for meat; on average 4 chickens per household Home gardens were not very common and often used for planting tobacco (40% of households). Only 6% of households planted mustard greens and only 2 months/year. Few households grew gourds or banana plants. Most families cultivated eggplants all year round in cassava or upland rice fields
Food sources	Phu Mo commune relied almost entirely on external food supplies. Apart from rice, 88% of households purchased foods for daily intake, mostly from mobile vendors. About 52% of households gathered wild foods from the forest, such as vegetables, mushrooms, and rats. Types of foods purchased from mobile vendors or markets in Dong Xuan district or nearby communes were: fish, meat and eggs (87% of households); vegetables (79%); sugar and salt (78%); rice, as their production was not sufficient (63%); and milk (30%) Local shops sold mostly dried and packaged foods such as instant noodles, porridge, and snacks
Dietary diversity	The local diet generally consisted of rice, cassava leaves, wild vegetables, chili, and salt Based on 24-hour recall, for all CU5 of Phu Mo commune (n = 243), most had consumed only one (24% of children) or two food groups (60%), predominantly grains. Less than 5% had a combined consumption of “starchy staples”, “meat and fish”, “fruits and vegetables”, and “dairy products”
School meals	Until the school year 2017-2018, none of the nursery schools in Phu Mo provided meals; in Dong Xuan district 15 of the 16 nursery schools in ethnic minority villages did not provide meals
School attendance	In the school year 2017-2018, average daily attendance was 88% (attendance is compulsory) but often only a few hours
On-going programs in the study area	At baseline, there were no Government or NGO programs/projects on-going or just completed. The NSA program was the first initiative in Phu Mo

Abbreviations: CU5, children under 5 years of age; NGO, nongovernmental organization; NSA, nutrition-sensitive agriculture.

The NSA program was implemented from 2017 to 2020, with the aim of reducing stunting and underweight among ethnic minority CU5 by improving food access and food intake. The NSA program involved three programmatic sectors—agriculture, education, and health—and multiple entry points—community, households, and nursery schools. The agriculture sector developed context-appropriate agricultural models aimed at supporting households with CU5 to plant nutrient-rich vegetables and fruit and raise chickens and quails, thus partially shifting from cash crops, to improve daily food intakes. The health sector trained household groups on nutrition, cooking, feeding, and monitoring growth of CU5 to facilitate behavioral change in child care and feeding. The education sector cooperated with local food micro-enterprises to develop the HGSF component. The latter is the focus of this manuscript and is described in more details below. Information about the overall NSA program in Phu Mo commune can be found in Box 1 and in a related publication.^41^

Box 1 NSA Program Description.

To address the undernutrition challenge among ethnic minority groups in Phu Mo commune, in 2017, the Medical Committee Netherlands-Vietnam (MCNV) in partnership with Phu Yen province and Dong Xuan district authorities initiated a four-year NSA program funded by the Netherlands Organization for Scientific Research with co-contribution of the local government. **The main aim was to reduce undernutrition by improving food access and food intake for children under five years of age (CU5)**. This action-research program was carried out with the scientific support of Hue University of Agriculture and Forestry, Hue University of Medicine and Pharmacy, and the Vrije Universiteit Amsterdam. Following the community empowerment approach used by MCNV in Dong Xuan district, the NSA program considered the capacity development among beneficiaries and local implementers as a key strategy. The training of the implementers at commune and village level was the primary responsibility of the district staff.

The **three sectors involved** in the NSA program were: **agriculture, health, and education**. All sectors contributed to the design and implementation of the integrated package of interventions, while each sector took responsibility for coordinating and monitoring specific components. Assessment of progress and improvement of design based on monitoring results was a joint undertaking. Undernutrition was addressed from different entry points: context-appropriate agricultural models (key entry point), health and nutrition education, and home-grown school feeding (HGSF).

***Agricultural component***—The main objectives were to improve homestead food production and promote a shift from cash crops to nutritious crops. Of 300 households with CU5 and/or pregnant women in Phu Mo commune, 150 households were targeted. Households with malnourished CU5 and those with more disadvantaged socio-economic conditions were considered eligible. Agricultural staff of the commune and beneficiary households received trainings on nutrition-sensitive production systems (which integrated poultry, fruit, and vegetables) in combination with in-field demonstration of sustainable land management techniques such as intercropping and agroforestry. A one-time small grant (US$ 172-229 depending on the complexity of the model) was offered to each household to establish the improved systems; the monthly income of these households is up to US$ 175. To facilitate the sharing of experiences, exchange visits were organized among the beneficiary farmers of the five villages involved in the NSA program.

***Health-nutrition component***—Focused on improving caring and feeding practices, including access to health services. All 300 households with CU5 and/or pregnant women participated, among them 150 households were also involved in the agricultural component. Training on early detection and treatment of severely malnourished children and pregnant women as well as training on nutrition counselling skills were provided to staff of the commune health stations and village health workers (VHWs). Local health staff was also trained on growth monitoring, disease prevention, hygiene, and breastfeeding practices. Practical trainings were also held at the community centers for the leaders of the twenty household groups in which the participating 300 households were organized. Such trainings capacitated the group leaders in the facilitation of the monthly household group meetings (HGMs) hosted in their homes. Communication material on relevant topics as well as measuring scales were given to the group leaders to facilitate the HGMs, which encompassed knowledge sharing on caring, feeding and health practices, cooking demonstrations, growth monitoring, and peer-to-peer support. Group leaders received a small compensation (approximately US$ 3.5/d) which was about 50% of their daily labor income.

***HGSF component***—Aimed to contribute to the program objective of reducing undernutrition in CU5 by supplying school meals to all five nursery schools in Phu Mo commune by 2020 and establishing sustainable local micro-enterprises for the preparation of the nutritious meals. All children registered in Phu Mo nursery schools were targeted. District and commune staff invited the few female small shop owners in the five villages to discuss the project and identified the most interested and competent. Four female micro-entrepreneurs, with business propensity and good cooking skills, were selected and trained by district staff of the Department of Education and Training on food preparation, hygiene and safety practices, and financial management.

The main objective of the HGSF intervention component was to reduce undernutrition among nursery school children (48-59 months old) from 30% in 2018 to 10% in 2020 by providing nutritious school meals, prepared using partly locally produced food, to the five nursery schools in Phu Mo. Through the HGSF component, the NSA program made efforts to change the supply chain of nutritious foods in these mountain villages by stimulating local production and demand. Four local micro-enterprises were established to prepare the meals; one covered schools in two nearby villages (Phu Giang and Phu Loi), while the others served the remaining three schools. The micro-entrepreneurs were all village women who could cook and wished to trade in nutritional products for nursery schools and the community. These skilled, motivated, and local micro-entrepreneurs were trained by the Health Centre and the Department of Education and Training to build good knowledge and practice on food preparation, hygiene and food safety, and financial management. To overcome the initial barriers to start-up their business, but also as an incentive, micro-entrepreneurs were provided with basic facilities, such as refrigerators, food containers, blenders, and rice cookers. Financial support to purchase the ingredients was provided only during the two-week training period before supplying school meals. Menus were based on the national school menu guidelines issued by the Ministry of Education, adapted by the District Education and Training Department to fit local factors, such as seasonality, availability of ingredients, food preferences, and culture. The menus were developed in two versions, for summer and winter, and for one month, with repetition in first and third and in second and fourth weeks. The nursery school head provided the planned menus to the micro-entrepreneurs. Seasonal or unavailable components could be replaced; the micro-entrepreneurs informed the head of the nursery school and the meal composition was adjusted to ensure a balanced diet. Only marginal changes occurred such as replacing chicken eggs with duck eggs. The micro-entrepreneurs relied upon a mixture of home-grown (own production and local farmers) and external (district market and mobile vendors) sources to procure the ingredients; the choice was based on local availability, food safety, and price. Food bought locally was generally cheaper than that purchased from external sources.

To ensure safe feeding practices, nursery school teachers received training on nutrition, feeding, and water, sanitation and hygiene (WASH) practices by the Department of Education and Training. In turn, teachers trained the mothers volunteering to help with feeding and caring for children on these topics, emphasizing hand-washing and cleaning utensils. The overall monitoring of the HGSF intervention was the responsibility of the District Education Department, the nursery school principal, and the teachers. Each nursery school assigned one teacher to monitor the appropriate supply of the daily menus and the correct substitution of unavailable ingredients. Confirmation of adherence to the planned weekly menu posted in the nursery schools also came from the parents. Regular meetings were organized among micro-entrepreneurs, teachers, parents, and program implementers to discuss progress and challenges regarding the school meals.

**Figure 1.**
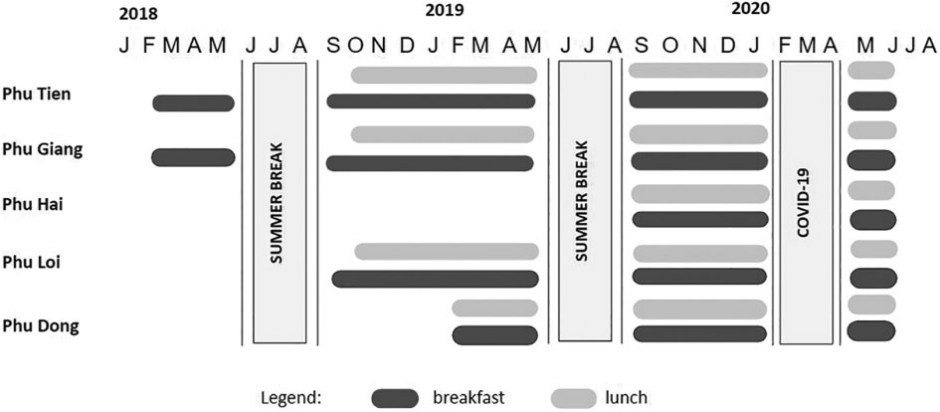
Implementation timeline of HGSF component in Phu Mo villages. HGSF indicates home-grown school feeding.

Nursery school children received school meals on weekdays, starting with only breakfast, from March 2018. However, the staggered entry program design meant that not all schools started providing meals in the first year (Figure 1). It should be noted that from September 2019, each child also received 180 mL of milk three days/wk under a school milk program launched by the provincial government—an unforeseen but valuable addition to boost their nutritional status.

To promote community buy-in, the NSA program fully subsidized a one-month pilot (about US$ 0.65 per child/d), after which payments were introduced, at first 33%, increasing to 50% from January 2020; from 2021, parents would cover the full cost. For the poorest households (at or below US$1.90 per capita/d), the government subsidizes 149 000 VND (about US$ 6.43) per month for school meals (45% of costs) and 100 000 VND (about US$ 4.31) per month for school supplies; the subsidy is disbursed twice a year. Teachers received an allowance from the project for their extra work (500 000 VND/month; about US$ 21.51). The micro-entrepreneurs earned about US$ 4.30/d for their labor, which was covered by the price of the school meals.

### Study Design

A mixed-methods assessment of the HGSF intervention with a focus on qualitative data collection provided complementary data on the observed and perceived changes brought about by the intervention, as reported by study participants. It included assessment of diversity and costs of planned school meals as well as an analysis of food sources used to gauge the extent of the “home-grown” dimension of the intervention. Process-related aspects were reviewed using qualitative methods, focusing on changes triggered by the HGSF intervention, observed barriers and facilitators, and perceived challenges and potential strategies to sustain the intervention after the NSA program. In the framework of this study, ***observed changes*** refer to key outcomes, such as increased school attendance, that in the perception of the respondents stemmed from the HGSF intervention together with the other NSA program components. The terms ***facilitators and barriers*** refer to those factors that enabled and/or constrained the implementation of the HGSF intervention. Such factors are usually related to the socio-economic, cultural, and bio-physical context in which the interventions are implemented. Contextual factors are less easily influenced and, in the context of an intervention, are often considered as a precondition. However, the level of influence that implementers have on these factors increases if they are internalized as part of the program design, for example, the role models and change agents in this case. The overall methodological approach for this study was largely based on the ex-post evaluation of a HGSF pilot program in Nepal by Shrestha et al^30^ and documented experiences with theory-driven process evaluation.^42^

**Figure 2.**
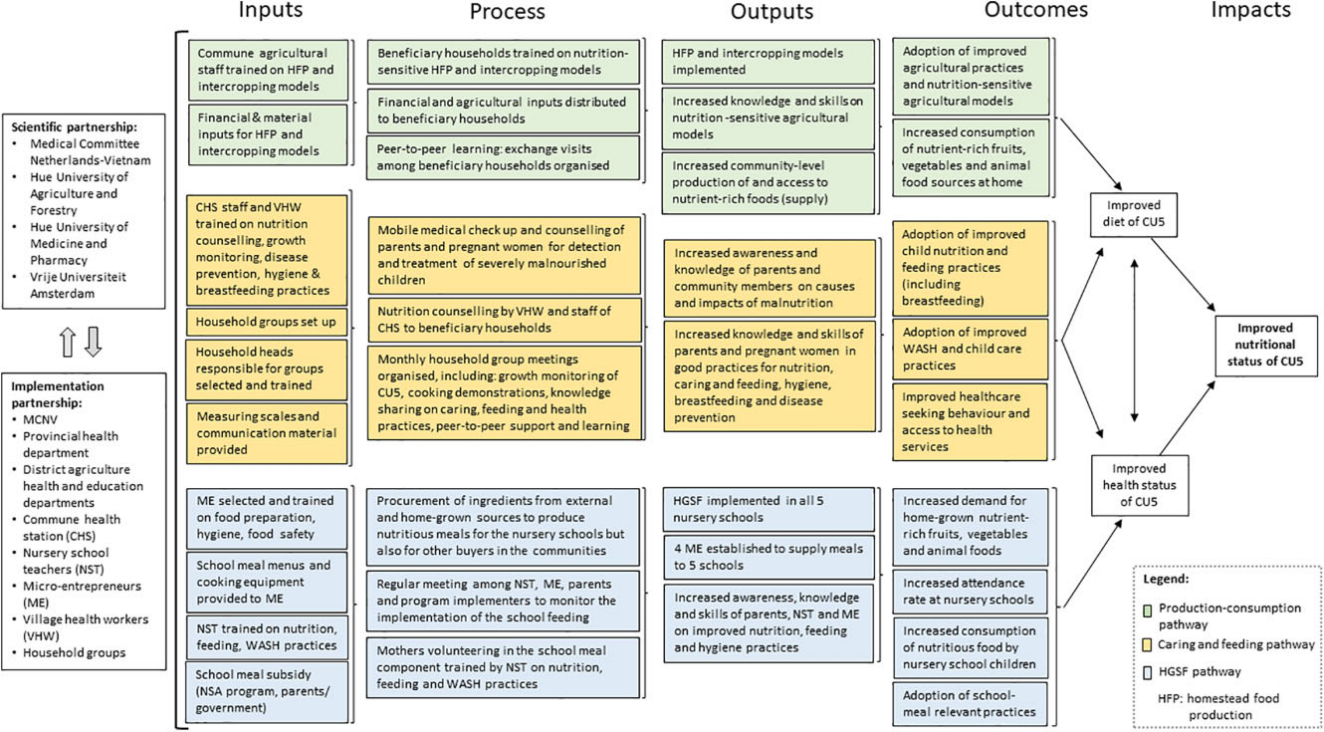
Ex-ante program theory of change.

The foundation for the qualitative research was an ex-ante program theory of change re-constructed using internal reports of the NSA program and other secondary sources (Figure 2). Its design was based on well-known conceptual pathways from agriculture to nutrition.^43,44^ The program theory allowed us to visualize and unpack the primary pathways of change envisaged by the program (production-consumption, caring and feeding, and HGSF) and to uncover the synergies emerging across pathways. The program theory informed the design of the data collection tools and the qualitative analysis. The study was approved by the Institutional Ethics Committee of Hue University of Medicine and Pharmacy in Vietnam (registration number H2018/010).

### Study Population

The direct beneficiaries of the HGSF program were the children (48-59 months old) attending the five nursery schools of Phu Mo. In the school year 2019-2020, 132 children were registered. In previous school years, the numbers were slightly lower (116 in 2018-2019 and 118 in 2017-2018; Ministry of Education data). Each year, around 45% of the children registered are attending the nursery school for the first time.

To strengthen the validity of the qualitative results, the process assessment included a variety of respondents from several stakeholder groups at different administrative levels: micro-entrepreneurs, teachers, parents/farmers in the villages, and implementers from the agriculture, education, and health departments at three levels. No interviews were done with children. See Table 2 for a complete overview of respondents involved and tools used. All five villages of Phu Mo commune (Phu Tien, Phu Hai, Phu Giang, Phu Dong, and Phu Loi) were included in the thirty semi-structured interviews (SSIs) and seven focus group discussions (FGDs; 76 participants in total). Purposive sampling was used to select the respondents, with the aim of obtaining views from all types of stakeholders. The main eligibility criterion for implementers invited for the SSIs and FGDs was to have knowledge of and be involved in all three components of the intervention package (agriculture, health-nutrition, and education). Only beneficiaries from households with CU5 and/or pregnant women were eligible for the study; the village leaders selected and invited them. For the FGDs at village level, parents were invited, but in fact only women attended, 9 to 18 per village. Written informed consent was obtained from all individual participants included in the study.

**Table 2.** Overview of Respondents and Data Collection Tools.

Level	Respondents	SSIs	FGDs	FGDs participants
Implementing NGO	Chief of office and lead implementer	1	–	–
District	Lead implementers from agriculture, health, and education departments	3	1	7
Commune	Principal of Phu Mo nursery schools, health station representative, agriculture extension staff	3	1	5
Village	Village health workers (VHWs)	5	–	–
	Nursery school teachers	5	–	–
	Micro-entrepreneurs	4	–	–
	Parents/farmers (across the five villages)	9	5	64
Total		**30**	**7**	76

Abbreviations: FGD, focus group discussion; NGO, nongovernmental organization; SSI, semi-structured interview.

### Data Collection and Analysis

#### Assessment of school meal diversity

In the context of this study, diversity was used as a measure of quality of the meals planned for the nursery school children. Breakfast and lunch menus were categorized into nine food groups, according to the Food and Agriculture Organization (FAO) Guidelines for Measuring Household and Individual Dietary Diversity,^45^ as used previously in Asia.^46^ The nine food groups were: starchy staples; dark green leafy vegetables; vitamin A rich fruits and vegetables; other fruits and vegetables; meat and fish; eggs; organ meat; legumes, nuts, and seeds; and dairy products. The Department of Education and Training provided the weekly summer and winter menus, which included the ingredients for each meal, and the quantity of each ingredient needed for the number of children to be served at each school. For the school meal diversity assessment, two alternating weekly menus, adapted for summer and winter, were scored separately and average percentages of food groups were calculated. *Inter-food group diversity*, the number of food groups, was assessed by assigning one point to any individual food item in each food group in one meal (breakfast or lunch). Different individual food items in the same group were not counted again. School meal diversity scores ranged from 0 to 9 (higher score = greater diversity). The results of the inter-food group diversity were then compared with the baseline 24-hour recall for CU5 in the NSA program (2018) to gain insights into the contribution of school meals to dietary diversity among nursery school children, irrespective of the quantities consumed. The *intra-food group diversity*, the number of unique individual foods within a food group, was also calculated separately for breakfast and lunch meals.

#### Assessment of food sources

Data on the sources of food used to prepare the meals were collected from each micro-entrepreneur in July 2020. Information was gathered on the percentage of “rice”, “vegetables and fruits”, “fish, pork and beef”, “chicken and quail eggs” and “chicken meat” procured from local farmers, own production, mobile vendors, and the district market. The first two sources were combined in the category “home-grown”, while the latter two were categorized as “external”. An overview of food categories per source was developed for each village and all villages combined.

#### Assessment of school meal costs

Data on school meal costs were provided by the Department of Education and Training at district level. They included the cost per ingredient for each breakfast and lunch on the planned menu, gas for cooking, and the cost of the micro-entrepreneurs’ cooking services. These costs were cross-checked with the actual expenses reported by the micro-entrepreneurs and their earnings for their labor. The cost of training micro-entrepreneurs and teachers was not included. Transportation costs were minimal as the micro-entrepreneurs were located near the schools. The average cost per meal per child was calculated by dividing the total meal cost (summer and winter meals separately) by the number of actual feeding days, and the average number of students attending the nursery school.

#### Qualitative process assessment

Qualitative data were collected between April and July 2020 using tools developed in English, translated into Vietnamese and checked for cultural appropriateness by staff of the Medical Committee Netherlands-Vietnam (MCNV). Semi-structured interviews (SSIs) and focus group discussions (FGDs) were conducted by a facilitator experienced in participatory data collection, with the assistance of a District Health staff member. Due to coronavirus disease 2019 (COVID-19) restrictions, some interviews were conducted remotely and video-recorded. All FGDs and the majority of the SSIs at village level were conducted in person and audio-recorded. Written informed consent and permission to record interviews were obtained from each respondent. Data from SSIs and FGDs were first transcribed verbatim into Vietnamese then translated into English. The quality and fidelity of transcriptions were checked by two local members of the research team against the audio/video records.

Semi-structured interview guides were designed using themes relevant to the ex-ante program theory of change (Figure 2) and taking into account elements of social entrepreneurship intention models, such as attitude towards becoming a social entrepreneur, self-efficacy, emotional empathy, and social support,^47^ for aspects related to the micro-entrepreneurs. The SSI guides were tailor-made for each stakeholder group and addressed process (changes resulting from the HGSF intervention, perceived barriers and facilitators) and sustainability-related aspects (challenges and strategies). A pilot test of the interview guide was run to improve fitness-to-purpose and to minimize duration. Interviews lasted approximately 60 minutes on average. They were organized in an iterative process; starting with district level, then commune and village level, while results were continually integrated with previous insights. Semi-structured interviews continued until saturation of data was reached and no new information emerged.

Focus group discussions were scheduled after all SSIs were completed to validate preliminary findings, to obtain deeper insights into frequently appearing themes, and to gain further lessons from different perspectives. The FGD tools consisted of open-ended questions and visual prompts to facilitate interaction. For example, the commune and district level tool used the ex-ante program theory of change to validate/refine the primary pathways (Figure 2) and identify synergies across pathways. It also helped stimulate intersectoral discussion on outcomes of the interventions, barriers and facilitators to implementation, home-made solutions to address barriers, and strategies to sustain interventions, with specific focus on HGSF. A simplified version of the tool was used for the FGDs at village level, where real-life pictures from Phu Mo facilitated discussions on intervention activities, perceived changes, how and why they occurred, and factors influencing those changes. Special emphasis was given to challenges and strategies for HGSF sustainability, especially regarding inclusion of the most vulnerable households. Focus group discussions at village level lasted about 60 minutes and involved parents whose children benefited from the school meal program; some were also farmers supplying food to the local micro-entrepreneurs. Focus group discussions at commune and district level had fewer participants and lasted longer (1.5-2 hours).

Several strategies were implemented to enhance the validity of qualitative results: notes of the SSIs and FGDs were taken and, if time allowed, verified with the respondents after each session. Interview summaries were used for member checking while district and commune meetings helped with collective validation of FGD records. The results of the qualitative process assessment were triangulated with reports of the field observations and transect walks conducted regularly (every month except during the COVID-19 quarantine period) by MCNV staff and district staff from agriculture, health, and education departments.

The English transcripts were summarized, coded, and analyzed using Atlas.ti 8.4.4 Mac. The ex-ante program theory of change (Figure 2) helped to structure the horizontal content analysis. A codebook was created through an iterative process; the central themes were identified deductively while the code list was generated inductively. The thematic analysis was informed by the principles for qualitative data analysis by Bazeley.^48^ Initially, one of the researchers systematically coded the SSIs and FGDs, then the other authors reviewed the original data and discussed code attribution to reach consensus. While the reported results reflect the perspectives of the different categories of respondents, specific quotes from individuals are used to illustrate key points.

## Results

In the first part of the results, we analyze the meals served to the children and the sources from which the ingredients used to make them were procured. In the second part, we describe the most important changes resulting from the intervention, the perceived barriers and facilitators, future challenges, and strategies expressed by different stakeholders during the SSIs and FGDs.

### School Meal Diversity

**Figure 3.**
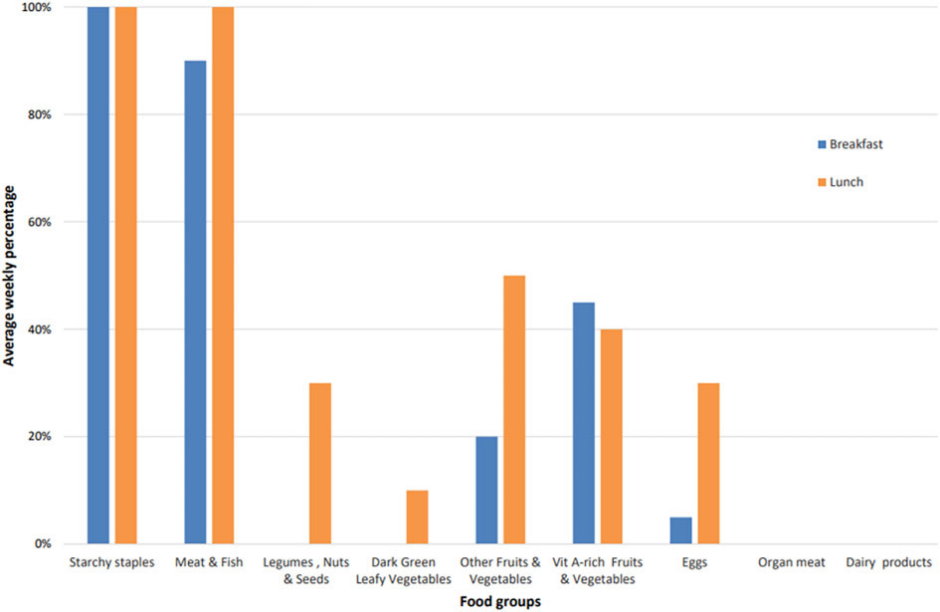
Inter-food group diversity—average percentage of food groups included in weekly school meals.

Four weekly menus were analyzed. In terms of *inter-food group diversity*, on average, a lunch menu contained 3 to 4 food groups and a breakfast, 2 to 3 (Figure 3). “Starchy staples” and “meat and fish” were the most frequently consumed food groups at both meals in both summer and winter. As for *intra-food group diversity*, lunch menus also had greater diversity within each food group, containing on average one more individual type of food per group than breakfast. For example, while lunch menus included chicken, shrimps, and pork from the group “meat and fish”, breakfasts comprised only chicken and shrimps. The greater inter- and intra-food group diversity of lunches was partly attributed to local culture, which considers lunch the most important meal of the day. Minimal differences were found in school meal diversity between the summer and winter menus. The school meals contributed to increasing the diversity of food consumed by nursery school children compared to baseline data (Table 1).

### School Meal Food Sources

**Figure 4.**
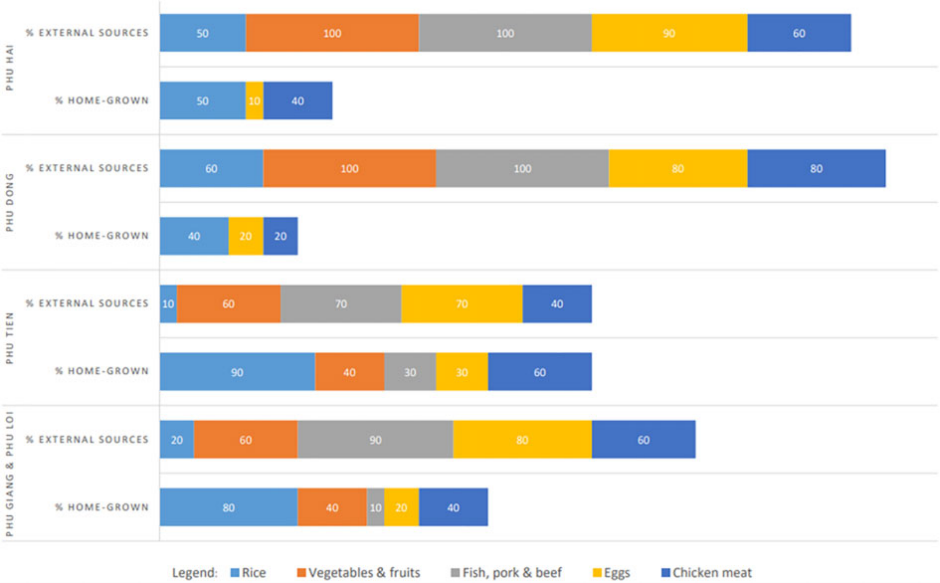
Overview of food sources for Phu Mo school meals.

The overview of food sources used by micro-entrepreneurs to procure ingredients for school meals in each village is presented in Figure 4. The “home-grown” category includes food obtained from local farmers and micro-entrepreneurs’ own production, while external sources are mobile vendors and the district market. The microentrepreneurs supplying meals to Phu Tien and the adjacent villages of Phu Giang and Phu Loi used home-grown food the most. Similarly to the situation at the onset of the NSA program, rice was most frequently obtained from home-grown sources (65% on average across all villages). Thanks to the increased local availability of nutritious foods, the “home-grown” dimension of the school feeding component grew larger over the program duration. On average, 31% of the foods used for school meals were home-grown, reflecting procurement choices of the two micro-entrepreneurs catering for Phu Tien, Phu Giang, and Phu Loi. As of July 2020, on average 40% of chicken meat and 20% of eggs were obtained from local farmers. Furthermore, the same two micro-entrepreneurs reported purchasing 40% of vegetables and fruits from home-grown sources. The foods least frequently obtained from home-grown production were fish, pork, and beef; in Phu Hai and Phu Dong, they were bought exclusively from external sources.

### School Meal Costs

The estimated total school meal cost was about VND 15 000/child/d (US$ 0.65), equivalent to 10% of the daily payment for hired farm labor. The average cost for breakfast was VND 5 048 (US$ 0.22) during summer and VND 4 934 (US$ 0.21) during winter; lunches cost VND 10 000 (US$ 0.43) in both seasons. Parents paid a standard amount of VND 15 000/child/d (including any program subsidy) for the two meals. This price could be kept constant because the micro-entrepreneurs were willing to adjust the payment for their labor so that expenses and price charged for the school meals were approximately the same.

### Qualitative Process Assessment

In this section, we firstly present the most important changes observed among children, parents, and the community that, in the perceptions of the different stakeholders, were stimulated by the HGSF intervention. Secondly, we report the perceived barriers and facilitators encountered in the implementation process. Thirdly, we describe the perceived future challenges to HGSF and strategies put forward by the respondents to overcome them. Lastly, an overview of perceptions across stakeholders is provided. While the different stakeholders were in general agreement about the observed changes, barriers and facilitators, and future prospects, each group had their own vision according to their role in the program.

#### Observed changes

An overview of the changes resulting from the HGSF intervention as reported by the different respondents is synthesized in Table 3. The most important changes described among nursery school children were their increased school attendance and an improvement in nutritional status. Attending school more regularly had an impact on meal frequency and diversity and appeared to have a strong influence on children’s food preferences and hygiene practices. Seeing the results of the intervention, in terms of children’s improved nutritional status, was crucial in motivating parents to change their caring, feeding, and hygiene practices. Furthermore, they became more willing to support the school feeding program and the micro-entrepreneurs. Community-wide changes relevant for the sustainability of the HGSF intervention were also observed. They concerned the strengthening of the local food system through a more stable supply and demand of home-grown nutritious foods, increased knowledge sharing, and building social capital.

**Table 3.** Overview of Changes Observed Among Nursery School Children, Parents, and the Community.

	Changes	Brief description of the change	Illustrative quotes
***Nursery school children***	Increased attendance	– According to Phu Mo nursery school reports, children arrived on time for breakfast and stayed until after lunch. They stayed home only when sick.	Teacher (R8): “Children changed considerably since this model has been applied. They go to school on time and more regularly.”
	Food preferences and hygiene practices	– Children’s food preferences were influenced and their eating regime became more regular under teachers’ supervision; children were motivated by eating with peers. – Increased school attendance improved children’s hygiene practices, especially handwashing, beyond school routine.	Micro-entrepreneur (R14): “When children are at home, they do not eat much. Parents do not encourage them. At school, they are together with their friends, so they eat more.” Commune FGD (G2): “Also at home, children are now proactive in washing their hands before eating and after the toilet.”
	Dietary intake and nutritional status	– Nutritious school meals together with improved feeding and hygiene contributed to better dietary intake and nutritional status as reported by several respondents and validated through observation. – A perceived reduction in children’s undernutrition was indicatively confirmed by results of official quarterly growth monitoring by teachers. In 2018-2019, with school feeding fully implemented, aggregated underweight was reduced from 26% to 14% of children. In 2019-2020, underweight declined from 33% to 17% in the first quarter, before children had to stay home for COVID-19 quarantine.	Micro-entrepreneur (R13): “Before the school meals, children often went home from school at around 10.30 a.m.; the few who had some money would buy candies at local shops but most would eat leftover rice at their parents. They did not have real lunches.” District education (R2): “Since March 2018, we implement the school meal program and we have observed that the malnutrition status clearly reduced.” Parent (R10): “The child gains more weight when she joins the school meal, I think because they have good food intake and drink milk. In the past, she was 11 kg; since she joined school meals, I weighed her at 13 kg.”
***Parents***	Caring and feeding practices	– Children became agents of change in the household: their explicit request to eat food like school meals at home led to changes in parental practices. – Changes in parents’ caring and feeding practices resulted from synergy between the HGSF component and nutrition education activities. Monthly household group meetings increased parents’ awareness of causes and impacts of malnutrition and behavior change to address the problem. – Mothers gained better cooking skills from demonstrations encouraging use of locally produced nutritious foods, reinforcing synergy among NSA program components. – Improvements in WASH-related practices and hygiene in the household environment were reported and observed.	District education (R2): “When children came home, they told their parents about the good food at school. Parents started to be concerned about food intake, because through the school meal they recognise that their children really like the new food, so they try to learn to make the same new dishes at home.” Commune FGD (G2): “Before, parents fed breakfast to their child with anything, not necessarily nutritious food. For example, instant noodles, or even no breakfast, but now they care about nutritious breakfasts like nutritious porridge.” Teacher (R18): “Children are taken care of in a better way compared to the past. I almost cried when I first started teaching at the nursery school in 2013. At that time, most parents and elderly people did not pay much attention to sanitation and to their children.” District FGD (G1): “When people have knowledge on agricultural production and nutritious meals, they can cook healthy meals for their children. (…) When kids have school meals and like them, parents who recognise that will cook the same at home. (…) The health sector can provide more knowledge and skills through cooking classes. I think, the knowledge on caring and feeding children is related to three sectors not only health sector.”
	Willingness to pay	– Parents expressed greater interest in their children’s nutritional status and higher commitment to pay for school meals (as much as they could pay). – A slight but relevant change in spending habits was noted; parents prioritized school meals over other expenditures. – 80% of parents expressed willingness to continue paying for school meals when the pilot ends.	Micro-entrepreneur (R7): “Parents tend to rely on state support; fathers often spend money buying beer and so. But recently, there has been a minor change; they pay more interest to their children’s schooling, for example by buying breakfast for their children.” Parent (R16): “Among mothers, those who support school meals say that it is not much money and we should continue to pay for them. For those who are poor, I think, it is easier to accept when the project supports 50%. But if they have to pay 100%, it is quite difficult.”
***Community***	Strengthening of the local food system	– The HGSF component increased local demand for nutritional foods, strengthening the “home-grown” dimension. The synergy between the agricultural and the HGSF components of the NSA program was self-reinforcing. – The food system in Phu Mo changed from cash-crop-oriented and dependent on external sources, to a partially self-reliant nutrition-sensitive food system. – Community-wide, the increase in local production, partly resulting from HGSF demand, not only improved household food security and reduced food expenditures but also created an income opportunity when surplus was sold, as done by at least 15% of the households.	District agriculture (R3): “In the past, villagers only focused on planting cassava or working as farm labour to earn money that they used to buy food. Now, many households are also concerned about raising chickens for eggs and planting vegetables to improve their daily intake, and for other households in the community. (…) Before, villagers bought almost 100% of their food from outside, but now, thanks to the agricultural models, they produce nutritious food in their village.” Micro-entrepreneur (R7): “Rice is nearly 100% locally supplied. Around 30%-40% of chickens are bought from local people. About 20% of eggs are locally supplied. 50% of vegetables are provided by locals and motorcycle vendors.”
	Increased knowledge sharing	– Community meetings on school feeding and household group gatherings strengthened both relationships and peer-to-peer learning; participatory knowledge-sharing events increased awareness and support for HGSF – Knowledge sharing between local government and communities built trust and gave opportunities to improve intervention design without fear of failure.	Commune nursery school principal (R4): “The schools collaborate with the health and agriculture sectors and the local enterprise. Every month we meet to agree on the provision of school meals and monitor the quality and food safety of the meals. We also join the household meetings together with agriculture or health staff.”
	Social capital	– Regular meetings of teachers, parents, and micro-entrepreneurs increased social capital. Better communication, bonds, and trust among community members led to confronting undernutrition as a community. Synergy between HGSF and nutrition education further increased social capital, giving benefits beyond the schools. – Increased social capital was also observed in the closer relations between communities and district, as evidenced by villagers calling the district agricultural staff directly with technical questions or where and how to buy materials.	District education (R2): “One positive impact I see is the collaboration and relationships among the community. For example, when the children come home, they praise the food at school, which makes parents believe in the teachers and local micro-enterprise.” District education (R2): “Some parents whose children don’t go to school, hear from parents with children at school about the meals, so they buy food for their children from the local enterprises. This also brings change to their homes.”

Abbreviations: COVID-19, coronavirus disease 2019; FGD, focus group discussion; HGSF, home-grown school feeding; WASH, water, sanitation and hygiene.

As presented in Table 3, changes attributed to the HGSF component were closely linked with changes resulting from other program components, such as increased variety in agricultural production, increased knowledge and skills on child care and nutrition, and community interactions. For example, the increased demand created by the HGSF component helped to incentivize the production system, enhancing local availability of and access to nutritious foods. The synergy among program components was described by a district official:

Because the NSA approach addresses undernutrition through agriculture, the interventions contribute to each other, for instance, entrepreneurs buying locally produced food supporting the agricultural model, or children at home requesting the same kind of food they receive in the school meal program. (…) This also changes the behaviour of the parents at home. (…) When the parents recognise that the children have good food intake and their nutrition status improves, then parents also change their behaviour and pay more attention to home gardens or chicken raising. (R1, District health)

#### Facilitators

Several factors facilitated the positive changes observed among children, parents, and the community in relation to the HGSF intervention. The identified facilitators—role models and enhanced confidence, change agents including committed micro-entrepreneurs—all contributed to increase the beneficiaries’ motivation to change.

##### Role models and enhanced confidence

Individuals who benefitted from the intervention, whose children’s nutritional status visibly improved, acted as role models by sharing their success story with others during household group meetings or at schools. A commune representative reported:

At school, when a child looks healthy and well-developed, teachers will be asked about the parents of that child, because other parents would like to meet them. (R4, Commune nursery school principal)

Exposure to positive examples and the tangible benefits of the intervention gave confidence to other beneficiaries and motivated them to replicate good practices. Using their context knowledge, the NSA program implementers effectively integrated this mechanism into the implementation strategy, as a district official recounted:

We select a household or model with good capacity. For these ethnic minority people it should be quite easy; when they see someone in the community who has success, they are eager to learn. So we focus on positive examples for others to learn and change their behaviour. (R1, District health)

The gained sense of empowerment and increased self-confidence were reported by several stakeholders, including the Phu Mo school principal:

Because we successfully implemented the NSA program in Phu Mo, we should bring people from other communes to visit the school meal model and the agriculture nutrition model here. (R4, Commune nursery school principal)

The MCNV staff observed that the pride and confidence of the district government increased when they could scale-up the school meal model (from 5 to 15 nursery schools by September 2020) with their own funding and its success was recognized in the national media.

##### Change agents

The individuals in each community who actively promoted change were of three types. The first group comprised local people who could influence the parents. The active engagement of socially recognized individuals capable of influencing others was deemed effective and culturally appropriate and was planned at the onset to leverage community buy-in for the HGSF intervention. The Phu Mo school principal explained:

To convince the parents, besides the education sector, we also use the voices of different stakeholders, for example the women’s representative, the village heads and the elderly of the community. So in the meeting with the parents we invite these people, they come together to convince the parents. (R4, Commune nursery school principal)

Secondly, as previously mentioned, nursery school children also acted as change agents within their own families. When they requested their parents to cook dishes similar to those at school, they triggered improvements in home-cooking practices. The third type is the micro-entrepreneurs, described in detail in the next paragraph.

##### Committed micro-entrepreneurs

Selecting committed and ambitious local candidates with the most promising capacity to set up successful food micro-enterprises was part of the implementation strategy. Social entrepreneurship proved to be a crucial facilitator of several aspects of the program, beyond the HGSF component. In fact, micro-entrepreneurs supplied nutritious meals not only to nursery schools but also to others in the community, thus benefitting more children while expanding their income opportunities and increasing demand for home-grown nutritious foods. A village health worker (VHW) explained the extended benefits of the micro-entrepreneurs’ role:

Mothers of children not yet attending preschool buy porridge for breakfast, for both well- and undernourished children, bringing them nutritional food. (R9, VHW/parent)

Furthermore, micro-entrepreneurs facilitated changes in home cooking practices by demonstrating their recipes to others. Their main motivation was not money, but the commitment to their own communities and their ability to contribute to reducing undernutrition, as observed by MCNV and district staff and explained by one micro-entrepreneur:

I like it (school feeding program) very much. It’s not because we can earn a bit more money, but the most important thing is that we find joy in what we are doing, and we are happy when we see the kids look better and playful. (R13, micro-entrepreneur)

That their motivation was not money was confirmed by their actions, as most micro-entrepreneurs continued to serve meals to all children, even those who were unable to pay on time. A micro-entrepreneur stated:

If just 50% of parents pay for the school meal, we will continue to serve their children. We just think that it is a pity for the rest of them not to enjoy school meals while their peers do. For us, it is most important to find out how to help all children in this community to have access to nutritious school meals. (R13, micro-entrepreneur)

Micro-entrepreneurs’ commitment and ambition were reflected in their quest to improve the menus to fit better the needs and preferences of the children, parents, and teachers, but also their openness to feedback, as explained by a micro-entrepreneur:

I got complaints from parents and school children about the quality of the food. I learned from that and tried to improve it. My expectation is to serve good food, and I feel happy when they are satisfied. The parents pay weekly visits to supervise whether the food is hygienic. I am quite happy because the food is now good enough. (R7, micro-entrepreneur).

#### Barriers

Most respondents stated that the school meal was affordable and highly valued for the multiple benefits accrued by the children. However, they also acknowledged the existence of several poverty-related barriers to participation, namely the need for more economically disadvantaged households to seek paid labor outside the communities. Those households lacked time to participate in the program and were unable to pay for school meals on time. Furthermore, land and water scarcity constrained agricultural production particularly in one of the villages.

##### Lack of time

The limited financial capacity of the poorest and most vulnerable households was recognized as a critical barrier for their participation in the HGSF; observation by MCNV staff confirmed this problem. The barrier persisted in spite of the NSA program subsidies and the willingness of these households to contribute. Poor households often relied on waged labor that kept them away from home, often for extended periods. These households lacked time to care for their children and to participate in relevant program activities, such as voluntary feeding shifts at nursery schools, school and household group meetings, and home gardens. A commune representative reported:

There is a lot of poverty in this area, so the people focus on working in the field or as hired labour. That creates difficulties in taking care of their children and feeding them. (R6, Commune health)

##### Late payments

Since the most disadvantaged households relied almost entirely on agricultural revenues, their income fluctuated, with droughts and floods affecting both production and income. They often failed to pay for school meals on time, thus affecting program implementation:

In the community there are many households in a difficult economic situation, so sometimes they contribute money late, this also influences the project. (R2, District education)

This point was validated by MCNV and district staff observations. A teacher clarified that poor households are not unwilling to pay but often lack the finances to pay on time:

There is no question that parents love the school meal model, but they have trouble earning enough money to make payments. The period from September to December is the rainy season, when parents cannot earn enough money for daily living. (R8, Teacher)

Furthermore, several stakeholders confirmed that for poor households, it is challenging to manage money on a monthly or even yearly basis, such as subsidies for school meals and supplies received from the Government twice yearly. A micro-entrepreneur explained:

They are used to spending on a daily basis, spending whenever they have money, they are not used to saving for a month to pay for the school meals. Looking at the money to be paid for the school meals in a month, it seems too much for them. They could manage it if they pay around VND 10,000 to 15,000 per day, they might find that affordable. (R13, micro-entrepreneurs).

##### Limited agricultural resources

Although production was constrained by land and water scarcity especially in the dry season in all five villages, one village found it more challenging to cope with these barriers and could hardly produce the surplus needed for the HGSF intervention, also in the rainy season.

#### Perceived future challenges and suggested strategies

With the end of the NSA program in sight, respondents identified critical challenges for the continuation of the HGSF intervention and outlined potential strategies to sustain it. Customization of payment methods to facilitate the participation of the most disadvantaged households, becoming semi-boarding schools, and strengthening the social entrepreneurship component were among the options proposed.

#### Challenges

##### School meal funding

In the framework of the NSA program, the school meals were subsidized at a decreasing rate; from 2021, their cost would be entirely covered by the parents. However, during implementation, it became evident that the poorest households struggled to remain engaged in school feeding, even with program support and their bi-annual government subsidy. According to most respondents, once external support ends, the majority of the households would probably be willing and able to continue paying for school feeding. However, the very vulnerable households, whose children would benefit most from the nutritious school meals, would probably drop out.

##### Limited resilience to shocks

Despite their remote location, Phu Mo communities were not exempted from the impact of the COVID-19 pandemic. In upland villages, food supplies and inputs for farming from outside became limited, hired labor jobs disappeared, and sales of agricultural products were delayed, directly affecting jobs, income, and food availability. For three months (February-April 2020) all program activities were suspended including household group meetings, trainings, and counselling activities. Schools and shops were closed, and children stayed home. The negative impact was immediately visible. By the end of the quarantine, the proportion of underweight children was almost as high as at the beginning of the school year. As a teacher recounted:

Some students were only 9 kg when they enrolled in my class but after the first semester, they were 11 kg. When the pandemic restrictions ended, children had lost weight and went back to their original weight. Now, children have to come back to school to start gaining weight again. (R18, Teacher)

District staff reported observing this situation during field visits following the quarantine. The COVID-19 pandemic was a serious setback for these poor communities. Nevertheless, it encouraged reflection on the importance of developing an even more self-reliant food system able to safeguard local food security and nutrition. It also confirmed the relevance of addressing undernutrition through multiple entry points (community, household, and nursery school). However, doubts were raised about the households’ capacity to continue applying the improved caring, feeding, and agricultural practices when challenges arise. Seeing the children lose weight during their absence from school could have indirectly contributed to increasing parents’ trust and support for the HGSF, potentially increasing its sustainability, as described by a VHW/parent:

I know this project helps improve children’s nutritional status. During the last three months of the epidemic, the children did not have sufficient nutritious food intake. Now the nursery schools open again. When I was there I saw the children eat eagerly. So, when the project finishes, I will continue to pay for the school meals for my children. (R9, VHW/parent)

The local authorities also expressed renewed support for the school feeding:

When the COVID crisis is over and the school is open again, the school meals will immediately start. I see no problem. (R2, District education)

#### Strategies

##### Financial sustainability of school feeding

Microentrepreneurs, teachers, and parents envisaged a number of strategies to address the barriers for the poorest households and sustain school feeding after the NSA program ends. Tailor-made payments (including alignment of the school meal payment with the disbursement of the government subsidy) and fundraising were among the solutions discussed in the district FGD:

First we will continue to convince the parents to maintain the school meal; second we will discuss with the local micro-enterprise on alternative payments, for example when the local micro-enterprise hires labour to harvest cassava or rice, they can hire these households. Or the micro-enterprise can wait until these families harvest their crops or earn money, then they can pay. (…) We can mobilise funding from charity groups in Phu Yen. (G1, District FGD)

Furthermore, a micro-entrepreneur explained in more detail her arrangement with the parents and the teachers:

In the last rainy season, some parents had no work and lacked cash to pay for school meals. So I talked to the teachers and told them to collect as much as possible, then give me the list of parents who still owed money; they can pay me later, when they have money. (R13, micro-entrepreneur)

The effectiveness of such customized payment arrangements has also been observed by MCNV staff.

##### Semi-boarding school

Many parents expressed a preference for a semi-boarding arrangement and extension to younger children. Children could take a nap at school after lunch, giving parents more time to work and raise income to pay for the semi-boarding arrangement. By September 2020, in fact, four of the nursery schools were providing this service for 113 children, as a follow-up to the school meal program.

##### Social support networks

Another strategy suggested to support the more disadvantaged households relying on waged labor outside the communities was to mobilize social support networks. Those parents could ask other community members to look after their children. This way children could still be adequately fed and have growth monitoring and healthcare like children from better-off households. This proposal not only showed stronger solidarity and use of social capital but also parents’ greater awareness of appropriate care and feeding practices, as remarked in the commune FGD:

There is a new behaviour of caring for the children; in the past, parents just went for work, but now if they work they try to find someone to help take care of their children. (G2, Commune FGD)

##### Social entrepreneurship

Respondents recommended investing more in the micro-enterprises because they meet community needs, are embedded in, and committed to, their communities, and are perceived to be sustainable. District agriculture staff observed that there had never been such local enterprises in the villages, where most food was brought by mobile vendors. Now, the role of the micro-entrepreneurs is recognized, as exemplified by their growing number, and should be supported. A district health representative also emphasized the importance of good collaboration between public and private sectors:

I think that the collaboration between private sector and local government within the NSA project is very good, but the local private sector plays the most important role because they live in the community. (…) I know from other projects, people from outside come to work and afterwards they go away and there are no results. So, for me, the investment in the locality is very important. (R1, District health)

##### Reduced reliance on program support

From 2017 to 2020, MCNV provided financial and technical support to the NSA program and coordinated the multi-level/multi-stakeholder partnership. The potential risk that, at the end of the program, MCNV would leave a void difficult to fill, led several respondents to reflect on the program exit strategy and the way forward. They suggested that the district government should take over the coordinating and supporting role to sustain the collaboration and replicate the successful experience of Phu Mo in other communes. Respondents advised integrating the NSA approach into prospective government programs for the social and economic development of upland communities, and developing training materials to continue building the knowledge and capacity on NSA of all sectors involved.

#### Perceptions across different stakeholders

There was general agreement about the observed changes, barriers, and facilitators and future prospects for the HGSF intervention among the different stakeholders. However, each group had their own vision according to their role in the program. For example, district authorities focused more on changes in the local food system, while VHWs described improvements in healthy behaviors of parents and children. Micro-entrepreneurs focused on food sources and emphasized their central role in the program. Both parents and teachers praised the children’s acquired habits and food preferences. A synthesis of the different perspectives is presented in Table 4.

**Table 4.** Perceptions Summarized Across Different Stakeholders.

Stakeholder group	Perceived changes	Facilitators (F) and Barriers (B)	Future prospects
All	• Reduced undernutrition • Increased school attendance • Improved caring and feeding by parents • Increased social capital • Stated willingness to pay school meals	F: Role models, change agents, and knowledge sharing opportunities instrumental in process of change B: Poverty is the main barrier	• Willingness to maintain school feeding program • Limited ability to pay of the poorest families • Financial risks depending on government and other funding • Lack of resilience to shocks like COVID-19
District authorities	• Caring and feeding practices by families improved (health and education) • Local food system: more balance between cash crops and non-cash crops and increased food availability (health and agriculture)	F: Role models enhanced confidence (all) F: Influential people involved to educate others (health and education) F: Children request same food at home (health) B: Irregular cash income and lack of time (all)	**Challenges** • HGSF sustained if government keeps existing subsidy policies (education and agriculture) • COVID-19 halted HGSF implementation (education) • **Strategies** • Tailor-made payments, support from charity groups, for poorest households (all) • Social entrepreneurship: HGSF sustainable if micro-enterprises continue to thrive (health) • Reduce reliance on program support (health)
Commune authorities	• Parents have more knowledge on nutrition and more concern for children • Increased home-grown food supply • Increased food availability and food intake at household level • Shift from buying food with wages to producing own food and selling surplus • Household groups and school meetings enhanced social capital	F: Role models and participation of villagers from beginning built confidence F: Selection of active/committed micro-entrepreneurs F: Exposure visits for knowledge sharing B: Irregular cash income and lack of time	**Challenges** • Difficult for some parents to pay full cost • During COVID-19 undernutrition rates returned to previous high levels **Strategies** • Encourage parents’ contribution to sustain school meals • Semi-boarding school approach • Social support networks: community members help each other • Increase support for more local micro-enterprises • Request district to provide more training and materials on NSA
Village health workers	• Parents bring children to school more regularly • Children change eating behavior at school • Parents spend more money on food for children • Households with sufficient water have enough nutritious food for children • Household group meetings enhanced social capital	F: Role models enhanced confidence: seeing results motivates people F: Eating with peers motivates children B: Irregular cash income and lack of time B: Lack of knowledge and awareness	**Challenges** • 60% to 70% of parents are willing and able to pay the full cost of school meals but not the poorest Strategies • More training needed on NSA topics
Nursery school teachers	• Children attend school more regularly and on time • Children changed hygiene habits and food preferences • Parents spend more money on food for children • School meals are made using local food • Increased communication among teachers, parents, and micro-entrepreneurs	F: Micro-entrepreneurs monitor child needs and improve meals F: Micro-entrepreneurs provide alternative payment methods for poor families F: Teachers have opportunities to advise parents on children’s health, cooking nutritious food B: Lack of money (especially in rainy season) and time reduces parents’ participation B: Parents working away unable to take good care of children B: Some parents cannot pay for school meals on time	**Challenges** • Paying full cost of school meals will be a challenge • Home feeding not yet of sufficient quality and quantity (children lost weight during COVID-19 lockdown) • Teachers motivated by program support (allowance) **Strategies** • Semi-boarding school approach also for younger children; more time for parents to work • Need for government support
Micro-entrepreneurs	• Children changed food preferences • Parents more concerned about their children; some buy same meals for children not in nursery school • Parents are willing to pay for school meals, about half are able to • Local food sources are used for school meals, including micro-entrepreneurs’ own products (less diversity than at district market but safer and cheaper) • Increased trust in the capacity of micro-entrepreneurs	F: Micro-entrepreneurs committed to improve meals to fit children’s needs and to provide meals for all children F: Micro-entrepreneurs teach parents how to prepare meals B: Late payments by parents limit food procurement by micro-entrepreneurs B: Food supply challenges: high market prices B: Poor financial management: parents not used to save money	**Challenges** •COVID-19 increased child undernutrition and affected activities and businesses **Strategies** • Micro-entrepreneurs will continue serving meals and even expand in coverage and/or number and diversify their markets
Parents/farmers	• Parents have more spare time; pay more attention to children’s nutrition • Parents see that children like the food at school; they get good meals • Increased availability of nutritious food and change in food intake	F: Peer-to-peer meetings promote learning, knowledge sharing, and behavior change B: For some, lack of time and irregular cash income for regular payment of school meals	**Challenges** • All want to sustain the school feeding program but not all are able to pay **Strategies** • Arrange payment scheme with micro-entrepreneurs

Abbreviations: COVID-19, coronavirus disease 2019; HGSF, home-grown school feeding; NSA, nutrition-sensitive agriculture.

## Discussion

The results of our study show that an HGSF intervention in nursery schools in remote villages, providing nutritious and diverse meals using partly home-grown products, could improve children’s dietary diversity, school attendance, food preferences, and WASH practices also at home. There was a perceived improvement in parents’ caring and feeding practices and an increased commitment to HGSF; many parents expressed willingness to cover the full cost of school meals. Positive changes were also observed at community level. Stakeholders reported that, compared to the NSA program baseline situation, the local food system became less cash-crop-oriented and more self-reliant in production of nutrient-rich foods, contributing to enhanced food security and to livelihood when surplus was sold, some of it to the micro-entrepreneurs providing the school meals. An increase in social capital was also observed, with better communication, bonds, and trust among community members. These positive changes were attributed not just to HGSF on its own but to the synergy among the NSA program components. Crucial facilitators were enhanced confidence and motivation, especially among parents, thanks to positive influences of role models and change agents such as the micro-entrepreneurs. The most important barrier was poverty, which, along with the limited resources and low resilience to external shocks, poses serious challenges to sustainability. Custom-made payments for disadvantaged households, mobilization of social support networks, and increased investment in social entrepreneurship could be viable strategies to sustain HGSF, particularly in isolated areas.

### Children’s Nutritional Intake and School Meal Diversity

The main aim of the HGSF pilot in Phu Mo commune was to reduce undernutrition among preschool children by improving their nutritional intake through introduction of nutritious school meals. Stakeholders from all sectors as well as parents reported consistently that children’s nutritional intake had improved. In Phu Mo, the children had breakfast and lunch at school and the quality of the food consumed at home was reported to have improved under the influence of the NSA program. The composition of the planned school meals (from which actual meals differed only marginally) most likely contributed to the increased daily dietary diversity for the children from 1 to 2 food groups at baseline to 3 to 4 after intervention. Our results align with a study assessing dietary diversity of children 3 to 5 years old in a school nutrition pilot program in poor, ethnic minority communities in rural China^46^ and a study of a HGSF pilot program in Nepal.^30^ One noted difference was the almost total absence of animal proteins in the school children’s diet in the Nepal program as well as the Ghana School Feeding Program,^33^ whereas the inclusion of “meat and fish” in school menus was prominent in our study. The inclusion of meat and fish in the meals served in Phu Mo nursery schools aligned with the animal protein requirements of four- to six-year-old children in the Recommended Dietary Allowances for Vietnam.^49^ The limited presence of animal food sources observed in other studies could be explained by factors such as culture-related diet preferences, a focus on increasing micronutrient content using less expensive foods (legumes and green leafy vegetables), or local availability and affordability of animal food sources.^28^

Another reported effect of providing the school meals was that children were accompanied to school more frequently and on time for breakfast. This increased school attendance had a positive effect on children’s meal frequency, food preferences, and hygiene practices. These factors, combined with the beneficial effect of diverse and nutritious school meals contributed to the perception that children’s nutritional status improved. A mixed methods study on HGSF in Nepal reported similar outcomes in terms of increased school attendance, improved dietary intake and hygiene practices.^30^ Tette and Enos^23^ reported consistent findings of increased school enrollment with partial increases in attendance, punctuality, and retention.

In line with the findings of previous studies,^30,50^ our results confirm the importance of strengthening knowledge and awareness among parents, caterers, and school staff on the need to consume diverse and nutritious meals to have better nourished children. It also emphasizes the relevance of multiple entry points (community, school, and household) to improve children’s nutrition, health and well-being, and the need for a closer involvement of parents/caretakers and other community members in school-based programs. Similar conclusions, from a health perspective, were drawn from an NSA school-based project in Burkina Faso.^25^

### Food Sources and Influencing Factors

On average, above 30% of the foods used for the school meals in Phu Mo were home-grown. That the average reached this high level was predominantly because of the procurement choices of two micro-entrepreneurs catering for Phu Tien and the adjacent villages of Phu Giang and Phu Loi. The difference in home-grown procurement was largely explained by the availability of the promoted nutritious foods in those villages. The limited local availability of recommended foods, such as vegetables and eggs, as a constraint to full-fledged implementation of NSA interventions has been reported. Schreinemachers et al,^51^ investigating the impact of school gardens and nutrition education in Burkina Faso, observed that although children were taught the importance of vegetables for their health, vegetables were hardly included in school menus or home diets because of low availability.

*Limited agricultural resources*, such as land and water, constrain production and diversification in the poorest villages leading to a lack of the surplus produce needed for the HGSF. In such cases, NSA programs should consider a wide range of production models and/or alternative livelihoods to find the best fit to the context and the local capacity. Additionally, programs could internalize such barriers and allocate sufficient resources or consider co-location with rural development programs addressing structural barriers.^52^

Also, *seasonality* could affect HGSF by influencing food supply (availability and diversity) and prices,^15,28,30,33^ thereby determining the choice of food sources for the school meals. In Phu Mo, seasonal changes in rainfall might have justified differences in the menus. However, assessment of school meal diversity in winter and summer revealed minimal differences. It could be that, during summer, when local production decreases but road access improves, micro-entrepreneurs use more external sources to procure foods. This explanation aligns with FAO and World Food Programme^15^ recommendations on temporary substitutions to fill seasonal gaps in food supply to ensure a smooth continuation of HGSF. Storage, food processing, and preservation could offer other viable options^15^; these were not investigated here, considering the limited local production surplus but may become interesting options in the future.

Seasonal factors are foreseeable and can be accounted for, but natural disasters and *external shocks* are less predictable and could undermine the limited resilience of local food systems, with direct consequences on HGSF. At the time of our study, COVID-19 threatened both the people’s health and the local food system. In view of future shocks and to prevent disruptions in supply, strategies such as local production of agricultural inputs^52^ and diversification of production, including biofortified crops, need to be further prioritized.^53^ From the demand-side, HGSF could offer alternatives to ensure continued meals for students even during the COVID-19 pandemic, as was recommended for the Brazil National School Feeding Program.^54^ Strategies include mobilizing emergency funds and incentives for local food purchases, but also providing takeout lunches and/or food kits, particularly for disadvantaged families.

### Cost of School Meals

Despite its benefits, the success of HGSF implementation depends significantly on whether it is affordable,^30^ especially for the economically disadvantaged households that could most benefit. In the Phu Mo HGSF pilot, the school meals cost was about US$ 0.65/child/d or US$ 13 per month. Margolies et al^55^ calculated the complete cost of school meals for nursery school children in Malawi; the costs compared favorably with other integrated agriculture and nutrition interventions. A notable difference with our program was that in their case, the families contributed a substantial proportion of the foods used in the school meals. For the community in Phu Mo, the HGSF would be a sound investment, as recent estimates from a cost–benefit analysis in Lao PDR show that for every dollar spent in school meal programs, the economic return is between US$ 5 and US$ 6.1 over the lifetime of a beneficiary.^56^ Most respondents considered the school meal affordable and appreciated their benefits. However, as government subsidies covered 45% of the school meal costs for only the poorest households, the continuation of HGSF in Phu Mo after project subsidies end raises concerns, because key sustainability strategies envisioned by the local stakeholders, such as customized payments, rely heavily on the social support, flexibility, and financial capacity of the micro-entrepreneurs.

### Micro-entrepreneurs’ Motivation and Future Prospects

The continuation of HGSF depends significantly on the incentives for the local micro-enterprises to maintain and scale up activities. In the current cost structure, micro-entrepreneurs earn about US$ 4.30/d from the price of the school meals for their services, lower than the average daily wage for farm labor (US$ 6.5). The micro-entrepreneurs were willing to earn relatively little from the school meals; their personal motivation and commitment to their communities played a greater role than financial incentives. The female micro-entrepreneurs did identify added value from running their business: more regular work compared to hired labor, being located in their own villages, being able to combine work and household chores, and to mobilize family members for meal preparation and delivery when needed. The micro-entrepreneurs showed pride in their role and its social recognition as well as their contribution to address undernutrition in their communities. Respect, trust, social recognition, and appreciation for the work done were common denominators in other studies that noted the motivational factor among local human resources at the frontline of NSA projects.^57-59^

Although low earnings could be acceptable in the framework of a program, given the support provided, there are concerns about sustainability, as without higher margins, micro-entrepreneurs might not have enough money to replace or keep equipment and premises up to standards. Long-term sustainability may be achieved by broadening their range of activities beyond school meal catering. Also, as diversification and appreciation for their services increase, more micro-enterprises could be established thus decreasing the responsibility of and reliance on the few existing ones, creating opportunities for others and enhancing HGSF sustainability. The latest NSA program report (September 2020) informed us that there were already seven micro-enterprises in Dong Xuan, serving 12 nursery schools, providing meals for 340 children. Another 85 children received meals from kitchens established in three other nursery schools. This represents a significant change from before the pilot intervention, when only one of the 16 nursery schools in the district provided meals. Clearly the school meal model has been accepted and supported by the communities. The increased availability of home-grown nutritious foods facilitates that process.

### Change Agents and Role Models as Key Facilitators in the Process

For changes to occur, certain community actors can be more influential than others. Change agents and role models are common features in Vietnamese culture. This NSA program leveraged on these contextual features and effectively integrated them in the implementation strategy to stimulate positive changes. Our study shows how socially recognized authorities, such as village leaders and women’s representatives, but also others such as micro-entrepreneurs and school children, could act as facilitators in the change process. The importance of key influencers and change agents as part of the community mobilization and engagement strategy has been highlighted in other NSA studies.^60-62^ For example, Ogoye-Ndegwa et al^61^ described ways to empower primary school children to become change agents for community development in the context of a school-based nutrition action-research project in Kenya. In our study, nursery school children triggered changes in the home environment, a spin-off not envisaged in the ex-ante program theory. However, we concur with Erismann et al^25^ that although children can be effective at promoting messages received at school, the uptake and translation of these messages into actual behavior changes at home may be difficult and take time. Simultaneous actions at community, school, and household level could have accelerated the change process in Phu Mo, for example, by reinforcing messages through a multi-pronged communication strategy as reported in the process evaluation of other NSA projects.^44,63^

### Home-Grown School Feeding Synergy With Agriculture Component

Finally, our results suggest that in remote and underserved areas having less developed, cash-crop-oriented food systems, HGSF can show many benefits, if it is integrated in a broader NSA program that mobilizes the entire community and stimulates both supply and demand of nutritious food. In line with the theory of change underpinning HGSF,^32,64,65^ this pilot intervention showed that HGSF contributed to the transformation. Before the NSA program, Phu Mo commune produced mostly cassava and relied significantly on external food supplies; none of the nursery schools provided school meals. The declining returns from cassava production, identified at baseline, may have triggered a desire for change, creating a window of opportunity for NSA interventions. Similar to other studies,^30,33^ promoting direct supply linkages with local farmers contributed to the creation of a short supply chain through development of context-appropriate agricultural models and establishment of social micro-enterprises as key intermediaries for procurement and catering. To overcome initial supply constraints, program interventions boosted local farmers’ capacity (training, material inputs, technical assistance) as envisaged in previous studies.^27,65^ The design choice to implement the agricultural component prior to HGSF was instrumental, as the surplus production could partly fulfil the need for school meal ingredients. According to Olney et al,^66^ the impact of agriculture interventions on production and consumption can only be observed after sufficient time. The benefits of carefully sequencing the activities in NSA programs were highlighted by Nordhagen et al^67^; staggering of supply and demand sides was also suggested for data collection during monitoring.^42^ While stimulating the supply side was necessary, selling surplus food to the micro-enterprises and to other households occurred spontaneously, resulting from community dynamics rather than program incentives. In the context of our study, progress reports of the NSA program revealed that the self-reinforcing cycle of supply and demand for local foods generated visible benefits only in the second year of implementation.

## Limitations

Due to logistic and financial constraints, it was not possible to assess school children’s dietary diversity using a 24-hour recall as was done for the program baseline. However, respondents reported that the school meals contributed increased diversity to the children’s daily diet. Our quality assessment of the school meals was based on the planned menus, not actual meals. According to program reports, there were only minor differences between the two. No specific data were collected about price differentials across options for sourcing ingredients. The anthropometric data available were only those collected routinely using a Ministry of Education and Training protocol, the timing of which was not optimal to capture the full effect of the HGSF program and could therefore not be used for this study.

## Conclusions

Our study expands the knowledge base on HGSF by describing the results of a pilot in a remote, poor ethnic minority community with a high prevalence of undernutrition among preschool children. Lessons were learnt from the integration of the HGSF component in a multi-sectoral program that mobilizes the entire community and stimulates both supply and demand of nutritious food. Home-grown school feeding in synergy with the other program components generated a range of benefits directly for the children. The positive effects spilled over to the home environment and the community. The local food system became more self-reliant for the production of nutritious foods, contributing to HGSF but also to better livelihood and household food security. This experience confirmed the importance of tailoring interventions to the context in which they are implemented and the power of integrating context factors, particularly culture-related ones, in the program implementation strategy, to stimulate positive changes that can be more easily accepted, adopted, and sustained by local communities. In a scenario of declining external funds and increasing community ownership, it will be important for the local authorities to continue investing in social micro-enterprises and to ensure that local budgetary economies are not made at the expense of good quality meals and the educational experience that is crucial for children in these formative years. The encouraging results of the pilot suggest that this approach shows potential in Vietnam, but more rigorous evidence is needed to assess benefits and trade-offs of implementation on a larger scale and in other contexts.
